# Synthesis and activity study of novel N,N-diphenylurea derivatives as IDO1 inhibitors

**DOI:** 10.3389/fchem.2023.1222825

**Published:** 2023-06-20

**Authors:** Xi-Xi Hou, Zi-Yuan Wu, An Zhan, En Gao, Long-Fei Mao, Hui-Li Wang, Jian-Xue Yang

**Affiliations:** ^1^ Department of Pharmacy, The First Affiliated Hospital, and College of Clinical Medicine of Henan University of Science and Technology, Luoyang, China; ^2^ School of Nursing, College of Basic Medicine and Forensic Medicine, Henan University of Science and Technology, Luoyang, China; ^3^ School of Chemistry and Chemical Engineering, Henan Normal University, Xinxiang, China; ^4^ State Key Laboratory of Quality Research in Chinese Medicine/Macau Institute for Applied Research in Medicine and Health, Macau University of Science and Technology, Macau, China; ^5^ Henan Wanliu Biotechnology Co., Ltd., Luoyang, China; ^6^ UNC Hospital, Chapel Hill, NC, United States

**Keywords:** indoleamine 2,3-dioxygenase 1, 1,2,3-triazole, N,N-diphenylurea, molecular docking, inhibitor

## Abstract

Indoleamine 2,3-dioxygenase 1 (IDO1) has attracted much attention in the field of cancer immunotherapy as an immunomodulatory enzyme. To identify potential IDO1 inhibitors, a novel series of compounds with N,N-diphenylurea and triazole structures were synthesized. The designed compounds underwent organic synthesis, and subsequent enzymatic activity experiments targeting IDO1 confirmed their activity at the molecular level. These experiments provided validation for the efficacy of the designed compounds in inhibiting IDO1, compound **3g** exhibited an IC_50_ value of 1.73 ± 0.97 μM. Further molecular docking study further explained the binding mode and reaction potential of compound **3g** with IDO1. Our research has resulted in a series of novel IDO1 inhibitors, which is beneficial to the development of drugs targeting IDO1 in numerous cancer diseases.

## 1 Introduction

**FIGURE 1:**
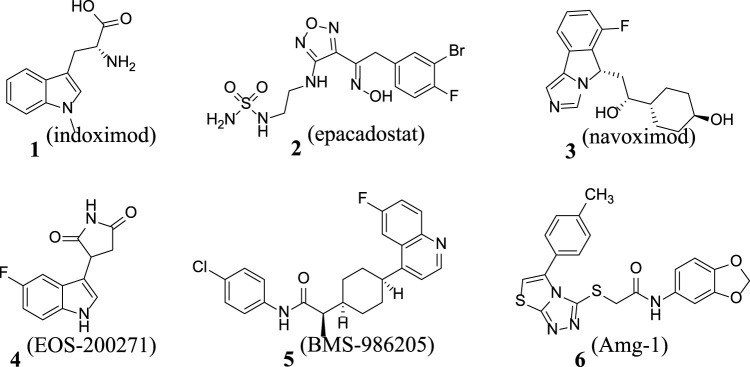
The chemical structures of six IDO1 inhibitors.

**FIGURE 2:**
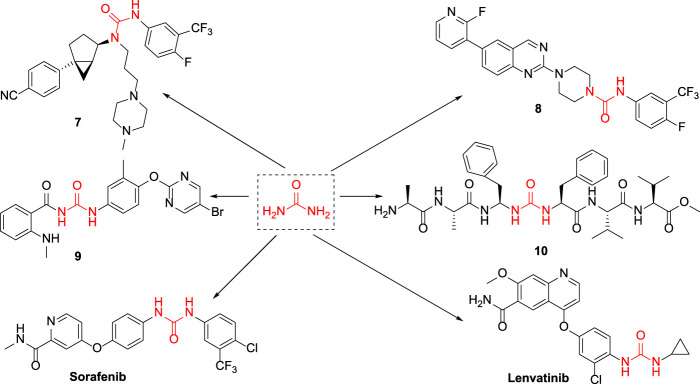
The chemical structures of six urea compounds.

Cancer is one of the malignant diseases seriously threatening human health. According to the estimates by the World Health Organization, there are approximately 14 million new cancer cases diagnosed globally each year (Sun et al., 2022). The number of cases continues to increase, and the new cases are expected to increase to 21 million in 2030. The mortality rate of cancer is extremely high, and nearly 10 million cancer patients die every year. In both developing and developed countries, cancer is one of the leading causes of death. Currently, despite many achievements in cancer therapy, some challenges remain. As technology advances, immunotherapy has become a research hotspot for cancer treatment (Serafini et al., 2020). There are various forms of immunotherapy, which include approaches such as T cells activated *in vitro*, oncolytic virus, and natural killer cells and using antibodies or recombinant proteins to co-stimulate cells or block the so-called immune checkpoint pathways (Röhrig et al., 2012). Through investigating the tumor immune microenvironment, IDO1 was found to be a metabolic enzyme that could mediate tumor immune evasion and a potent immunomodulator (Pan et al., 2020). It plays a role in pathogenic inflammatory processes and generates immune tolerance to tumor antigens, mainly through facilitating tryptophan consumption and producing a series of toxic kynurenine metabolites, thereby achieving immune tolerance by activating the GCN2 pathway, inhibiting the mTOR pathway and the toxic effects of kynurenine, and promoting the differentiation of regulatory T cells (Nelp et al., 2018). Presently, IDO1 is overexpressed in a variety of tumor tissues, and IDO1 has emerged as a potential target for studying tumor immunosuppression (Muller et al., 2005). Since 2015, multiple IDO1 inhibitor drugs have been tested in clinical trials, such as indoximod, epacadostat, navoximod, EOS-200271, BMS-986205, and Amg-1 (Kumar et al., 2019; Hou X. X. et al., 2022; Kapron et al., 2023; Wang et al., 2023; Ye et al., 2023) (Figure 1; Figure 2).

Urea is an organic compound that consists of carbon, nitrogen, oxygen, and hydrogen. In the 18th century, urea was first extracted from the urine of humans. In the 19th century, scientists found that the inorganic substance ammonium cyanate could be used as raw material to synthesize urea, which overturned the traditional vitalism theory and opened a new chapter in organic chemistry (Jr et al., 1993). Urea structural fragment is an important advantageous skeleton for drug development and is extensively used in the medical field (Nakagawa et al., 2009). For example, the melanin-concentrating hormone was associated with many physiological functions in the human body and played a pivotal role in especially the aspects of food intake and energy balance. Compound **7** with urea structure could be used as a melanin-concentrating hormone antagonist and is effective in reducing body weight (McBriar et al., 2006). Kotakadi et al. reported that compound **8** with urea structure had an excellent inhibitory effect on the proliferation of *Staphylococcus aureus* (Babu et al., 2015). Khanet al. discovered that compound **9** could effectively repress pancreatic cancer cells and laryngeal squamous carcinoma cells. The Lipton laboratory found that compound **10** exhibited a moderate inhibitory effect on HIV-1 protease. The currently marketed antineoplastic drugs containing urea structure include **lenvatinib** for the treatment of kidney or thyroid cancer, which is a tyrosine kinase inhibitor drug able to effectively slow down or terminate the growth of tumor cells (Zhang et al., 2020). **Sorafenib** is also included, which is a new multi-targeted oral drug for cancer therapy (Tang et al., 2020). It is used to treat gastrointestinal stromal tumors and metastatic renal cells that are not responding to standard therapy or are resistant, which could selectively target the receptors of certain proteins and is considered to act as a molecular switch during tumor growth (Yau et al., 2022). Its aforementioned indications have obtained the “fast track” designation granted by the FDA in the United States.

Considering the excellent biological activity performance of urea compounds, to find new and more efficient IDO1 inhibitor drugs, we aimed to link diphenylurea compounds with different substituents to triazole compounds, which were widely used in the modification of drug molecules (Rohrig et al., 2019; Qi et al., 2020; Zhang et al., 2020). Ten diphenylurea-linked 1,2,3-triazole derivatives were synthesized, these compounds were designed with the expectation of exhibiting specifict functions in inhibiting the activity of IDO1.

## 2 Chemistry

**FIGURE 3:**
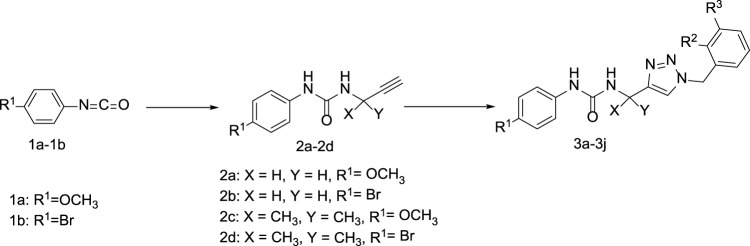
The reaction routes and the structures of compounds **3a-3j**.

Detailed operation as follows: The phenyl isocyanate derivatives (**1a-1b**) reacted with prop-2-yn-1-amine (or 2-methylbut-3-yn-2-amine) and triethylamine in the dichloromethane to form compound **2a-2b**. Compound **2a-2b** reacted with some azide derivatives to give compound **3a-3j** as shown in Figure 3 and Table 1. The molecular structures of these compounds were analyzed using ^1^H NMR and ^13^C NMR. Furthermore, we conducted additional testing to evaluate the anti-IDO1 activity of these ten compounds.

**TABLE 1:** R-group of compounds **3a**-**3j**.

Compound	X	Y	R_1_	R_2_	R_3_
**3a**	H	H	OCH_3_	Br	H
**3b**	H	H	OCH_3_	H	Br
**3c**	H	H	OCH_3_	H	OCH_3_
**3d**	H	H	Br	Br	H
**3e**	H	H	Br	H	Br
**3f**	CH_3_	CH_3_	OCH_3_	Br	H
**3g**	CH_3_	CH_3_	OCH_3_	H	Br
**3h**	CH_3_	CH_3_	OCH_3_	H	OCH_3_
**3i**	CH_3_	CH_3_	Br	Br	H
**3j**	CH_3_	CH_3_	Br	H	Br

## 3 Results and discussion

### 3.1 IDO1 inhibition study

Based on a comprehensive review of the literature, we employed a Hela cell-based functional assay to investigate the IDO1 inhibition activities of the designed compounds. (Table 2). We employed **Amg-1** as a positive control, whose IC_50_ value was measured to be 3.97 µM under our experimental conditions, which was close to the actual value (Hou X. et al., 2022). As shown in Table 2, the activities of compound **3a, 3c, 3g, and 3h** against IDO1 were all below 10 μM, of which **3g** reached 1.73 ± 0.97 μM. This indicates that some give synthesized compounds have good inhibitory activity.

**TABLE 2:** IDO1 inhibitory activities of designed derivatives.

Compd no.	IDO1 IC_50_ (μM)	Compd no.	IDO1 IC_50_ (μM)
**3a**	>20	**3f**	17.92 ± 3.14
**3b**	3.97 ± 0.92	**3g**	1.73 ± 0.97
**3c**	7.10 ± 1.48	**3h**	5.82 ± 2.65
**3d**	>20	**3i**	>20
**3e**	>20	**3j**	>20

IC_50_ values were fitted from single point inhibition curves, and two parallel experiments were performed for each compound. IC_50_ values were calculated using Graph Pad Prism 8.0 software. These results are reported as the averages ±SD.

### 3.2 Molecular docking studies of compound 3a, 3b, 3f, and 3g

**FIGURE 4:**
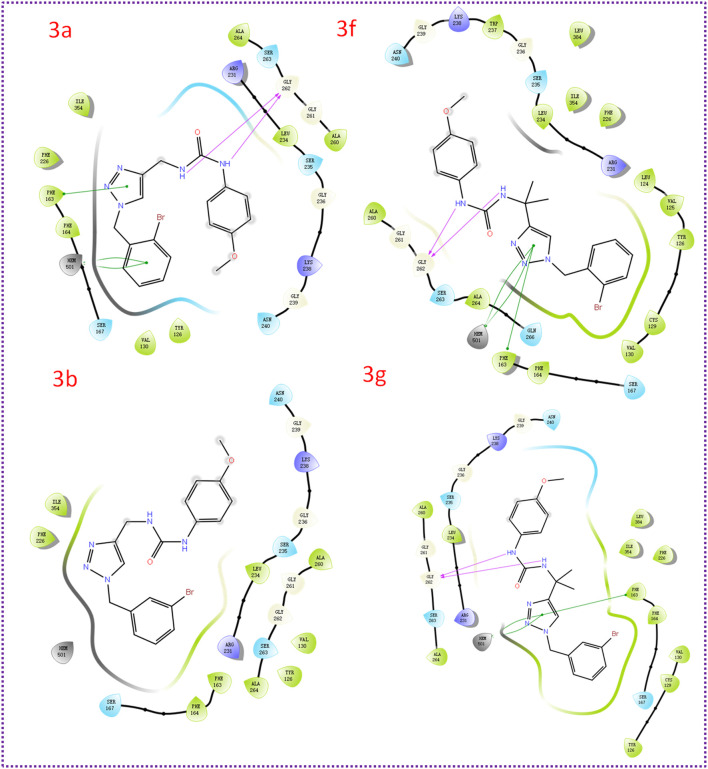
The binding modes of compound **3a**, **3b**, **3f, and 3g**.

To gain insights into the binding mode of the active compounds to IDO1, we selected compounds 3a, 3b, 3f, and 3g as model compounds. These compounds were chosen due to their varying levels of activity, allowing us to examine the potential differences in their binding interactions with IDO1. As shown in Figure 4, the docking scores of compounds **3a**, **3b, 3f** and **3g** with IDO1 were −6.424, −6.292, −7.665 and −6.876 kcal/mol, respectively. The 1,2,3-triazolyl group in the structures of compounds **3f** and **3g** were nearby the heme. And the binding mode of compounds **3f** and **3g** are similar to several reported IDO1 inhibitors. The 1,2,3-triazole group in the structure of compound **3a** and **3b** were far away from the heme iron and have weaker affinities for IDO1. The docking results indicated that compounds **3f** and **3g** were more capable of coordinating with ferrous ions than **3a** and **3b**.

## 4 Conclusion

Ten series of compounds that contain the N,N-diphenylurea structure and the 1,2,3-triazole structure were designed and synthesized. *In vitro* experiments were conducted to assess the compounds’ activity, and the results showed that the compounds with an isopropyl linker displayed better activity than those with a methyl linker. Notably, compound **3g** exhibited the most potent inhibitory effect on IDO1, with an IC_50_ value of 1.73 ± 0.97 μM. Further molecular docking studies confirmed that compound 3g is the most promising of the synthesized compounds.

## 5 Experimental protocols

### 5.1 Materials and chemistry

The 1,2,3-triazole derivatives were synthesized in-house by our research group. All reagents and solvents obtained from commercially available source were used without further treatment. ^1^H NMR and ^13^C NMR spectra were acquired in DMSO-d_6_ solution with a Bruker 600 spectrometer. Chemical shifts (d) were given in parts per million with tetramethylsilane as internal reference and coupling constants were expressed in hertz. Hela cell line was purchased from ATCC (Virginia). Recombinant human IFN-γ was purchased from R&D systems (Emeryville, CA). The 3.05 Ntrichloroacetic acid, was purchased from Sigma Aldrich (St. Louis, MI).

### 5.2 General procedure for preparation of compound **2a** (The method is suitable for **2b**)

To a solution of 1-isocyanato-4-methoxybenzene (1.5 g, 0.01 mol) in CH_2_Cl_2_ (100 mL) was added prop-2-yn-1-amine (0.6 g, 0.011 mol) in one portion. After stirring at room temperature for 1.0 h, the mixture was concentrated, and the residue was purified by column chromatography on silica gel (eluent: PE: EA = 5:1) to give compound **2a** as a solid.

### 5.3 General procedure for preparation of compound **3a** (The method is suitable for **3b**-**3j**)

The reaction reagents were compound **2a** (0.2 g, 1.0 mmol) and 1-(azidomethyl)-2-bromobenzene (0.2 g, 1.2 mmol), which were added to 15 mL of mixed solvent (water: THF: tert-butanol = 1:1:1). The reaction was performed in copper sulfate pentahydrate (0.1 mmol) and sodium ascorbate (0.2 mmol) at 80°C. After completion of the reaction, the mixture was extracted through CH_2_Cl_2_ (10 mL×3). The organic phase was combined and concentrated *in vacuo*. The desired compound 3a was isolated by column chromatography (CH_2_Cl_2_/MeOH = 20:1).

***1-*** ((***1-*** (***2-bromobenzyl***) ***-1H-1,2,3-triazol-4-yl***) ***methyl***) ***-3-*** (***4-methoxyphenyl***) ***urea*** (***3a***) ***.*** Gray solid. LC-MS(ESI): m/z 416.07 (M + H)^+^. Yield: 81.4%. ^1^H NMR (600 MHz, DMSO-d_6_) δ 8.34 (s, 1H, NH-H), 7.97 (s, 1H, NH-H), 7.69 (d, *J* = 6.0 Hz, 1H, CH-H), 7.40 (t, *J*_*1*_ = 6.0 Hz, *J*_*2*_ = 6.0 Hz, 1H, Ar-H), 7.32–7.27 (m, 3H, Ar-H), 7.14 (d, *J* = 6.0 Hz, 1H, Ar-H), 6.81 (d, *J* = 6.0 Hz, 2H, Ar-H), 6.47 (t, *J*_*1*_ = 6.0 Hz, *J*_*2*_ = 6.0 Hz, 1H, Ar-H), 5.66 (s, 2H, CH_2_-H), 4.32 (d, *J* = 6.0Hz, 2H, CH_2_-H), 3.69 (s, 3H, OCH_3_-H). ^13^C NMR (150 MHz, DMSO-d_6_) δ 155.7, 154.4, 135.5, 133.9, 133.3, 130.8, 130.8, 128.7, 123.6, 123.3, 119.9, 114.3, 55.6, 53.2, and 35.3.

***1-*** ((***1-*** (***3-bromobenzyl***) ***-1H-1,2,3-triazol-4-yl***) ***methyl***) ***-3-*** (***4-methoxyphenyl***) ***urea*** (***3b***) ***.*** Gray solid. LC-MS(ESI): m/z 416.07 (M + H)^+^. Yield: 89.7%. ^1^H NMR (600 MHz, DMSO-d_6_) δ 8.34 (s, 1H, NH-H), 8.08 (s, 1H, NH-H), 7.53 (d, *J* = 6.0 Hz, 2H, CH-H, Ar-H), 7.35–7.28 (m, 4H, Ar-H), 6.81 (d, *J* = 12.0 Hz, 2H, Ar-H), 6.47 (s, 1H, Ar-H), 5.59 (s, 2H, CH_2_-H), 4.31 (s, 2H, CH_2_-H), 3.69 (s, 3H, OCH_3_-H). ^13^C NMR (150 MHz, DMSO-d_6_) δ 155.8, 154.4, 139.2, 133.9, 131.4, 131.4, 131.2, 127.5, 123.7, 122.2, 119.9, 114.3, 55.6, 52.3, and 35.4.

***1-*** ((***1-*** (***3-methoxybenzyl***) ***-1H-1,2,3-triazol-4-yl***) ***methyl***) ***-3-*** (***4-methoxyphenyl***) ***urea*** (***3c***) ***.*** White solid. LC-MS(ESI): m/z 368.17 (M + H)^+^. Yield: 82.8%. ^1^H NMR (600 MHz, DMSO-d_6_) δ 8.34 (s, 1H, NH-H), 8.03 (s, 1H, NH-H), 7.29–7.26 (m, 3H, CH-H, Ar-H), 6.89 (d, *J* = 6.0 Hz, 2H, Ar-H), 6.86 (d, *J* = 6.0 Hz, 1H, Ar-H), 6.81 (d, *J* = 6.0 Hz, 2H, Ar-H), 6.46 (s, 1H, Ar-H), 5.53 (s, 2H, CH_2_-H), 4.31 (s, 2H, CH_2_-H), 3.73 (s, 3H, OCH_3_-H), 3.69 (s, 3H, OCH_3_-H). ^13^C NMR (150 MHz, DMSO-d_6_) δ 159.8, 155.8, 154.4, 138.0, 133.9, 130.3, 120.5, 119.9, 114.3, 114.2, 113.8, 55.6, 55.5, 53.1, and 35.4.

***1-*** ((***1-*** (***2-bromobenzyl***) ***-1H-1,2,3-triazol-4-yl***) ***methyl***) ***-3-*** (***4-bromophenyl***) ***urea*** (***3days***) ***.*** White solid. LC-MS(ESI): m/z 463.97 (M + H)^+^. Yield: 66.9%. ^1^H NMR (600 MHz, DMSO-d_6_) δ 8.71 (s, 1H, NH-H), 7.99 (s, 1H, NH-H), 7.68 (d, *J* = 6.0 Hz, 1H, CH-H), 7.40–7.36 (m, 5H, Ar-H), 7.31 (t, *J*_*1*_ = 6.0 Hz, *J*_*2*_ = 6.0 Hz, 1H, Ar-H), 7.15 (d, *J* = 6.0 Hz, 1H, Ar-H), 6.64 (s, 1H, Ar-H), 5.66 (s, 2H, CH_2_-H), 4.34 (d, *J* = 6.0 Hz, 2H, CH_2_-H). ^13^C NMR (151 MHz, DMSO-d_6_) δ 155.3, 140.2, 135.4, 133.3, 131.8, 130.8, 130.8, 128.7, 123.7, 123.3, 120.0, 112.9, 53.3, and 35.3.

***1-*** ((***1-*** (***3-bromobenzyl***) ***-1H-1,2,3-triazol-4-yl***) ***methyl***) ***-3-*** (***4-bromophenyl***) ***urea*** (***3e***) ***.*** White solid. LC-MS(ESI): m/z 463.97 (M + H)^+^. Yield: 90.3%. ^1^H NMR (600 MHz, DMSO-d_6_) δ 8.71 (s, 1H, NH-H), 8.06 (s, 1H, NH-H), 7.53 (d, *J* = 6.0 Hz, 2H, CH-H, Ar-H), 7.39–7.35 (m, 4H, Ar-H), 7.33–7.30 (m, 1H, Ar-H), 6.64 (s, 1H, Ar-H), 5.59 (s, 2H, CH_2_-H), 4.33 (d, *J* = 6.0 Hz, 2H, CH_2_-H). ^13^C NMR (150 MHz, DMSO-d_6_) δ 155.3, 140.2, 139.2, 131.8, 131.4, 131.4, 131.1, 127.5, 123.5, 122.2, 120.0, 112.9, 52.3, and 35.3.

***1-(2-(1-(2-bromobenzyl)-1H-1,2,3-triazol-4-yl)propan-2-yl)-3-(4-methoxyphenyl)urea (3f)***. White solid. LC-MS(ESI): m/z 444.10 (M + H)^+^. Yield: 77.2%. ^1^H NMR (600 MHz, DMSO-d_6_) δ 8.23 (s, 1H, NH-H), 7.99 (s, 1H, NH-H), 7.68 (d, J = 12.0Hz, 1H, CH-H), 7.38 (t, J_1_ = 12.0Hz, J_2_ = 12.0Hz, 1H, Ar-H), 7.30 (t, J_1_ = 12.0Hz, J_2_ = 12.0Hz, 1H, Ar-H), 7.23–7.20 (m, 2H, Ar-H), 7.06 (d, J = 6.0Hz, 1H, Ar-H), 6.78 (d, J = 12.0Hz, 2H, Ar-H), 6.40 (s, 1H, Ar-H), 5.65 (s, 2H, CH_2_-H), 3.68 (s, 3H, OCH_3_-H), 1.64 (s, 6H, 2CH_3_-H). ^13^C NMR (150 MHz, DMSO-d_6_) δ 154.8, 154.3, 154.2, 135.7, 134.0, 133.3, 130.7, 130.4, 128.7, 123.0, 122.3, 119.5, 114.3, 55.6, 53.2, 50.5, 40.5, and 28.9.

***1-(2-(1-(3-bromobenzyl)-1H-1,2,3-triazol-4-yl)propan-2-yl)-3-(4-methoxyphenyl)urea (3 g)***. White solid. LC-MS(ESI): m/z 444.10 (M + H)^+^. Yield: 82.4%. ^1^H NMR (600 MHz, DMSO-d_6_) δ 8.22 (s, 1H, NH-H), 8.05 (s, 1H, NH-H), 7.53 (s, 2H, CH-H, Ar-H), 7.36–7.29 (m, 2H, Ar-H), 7.23–7.20 (m, 2H, Ar-H), 6.78 (d, J = 18.0Hz, 2H, Ar-H), 6.39 (s, 1H, Ar-H), 5.57 (s, 2H, CH_2_-H), 3.67 (s, 3H, OCH_3_-H), 1.62 (s, 6H, 2CH_3_-H). ^13^C NMR (150 MHz, DMSO-d_6_) δ 154.7, 154.5, 154.2, 139.3, 134.0, 131.4, 131.4, 131.1, 127.4, 122.3, 122.0, 119.5, 114.3, 55.6, 52.2, 50.4, and 28.9.

***1-(2-(1-(3-methoxybenzyl)-1H-1,2,3-triazol-4-yl)propan-2-yl)-3-(4-methoxyphenyl)urea (3 h)***. White solid. LC-MS(ESI): m/z 396.20 (M + H)^+^. Yield: 87.7%. ^1^H NMR (600 MHz, DMSO-d_6_) δ 8.23 (s, 1H, NH-H), 8.01 (s, 1H, NH-H), 7.29–7.20 (m, 3H, CH-H, Ar-H), 6.89–6.77 (m, 5H, Ar-H), 6.39 (s, 1H, Ar-H), 5.52 (s, 2H, CH_2_-H), 3.72 (s, 3H, OCH_3_-H), 3.68 (s, 3H, OCH_3_-H), 1.63 (s, 6H, 2CH_3_-H). ^13^C NMR (150 MHz, DMSO-d_6_) δ 159.9, 154.7, 154.4, 154.2, 138.1, 134.0, 133.4, 130.3, 121.9, 120.3, 119.5, 114.4, 114.3, 113.9, 113.8, 55.5, 55.5, 53.0, 50.5, and 28.9.

***1-(2-(1-(2-bromobenzyl)-1H-1,2,3-triazol-4-yl)propan-2-yl)-3-(4-bromophenyl)urea (3i)***. White solid. LC-MS(ESI): m/z 492.00 (M + H)^+^. Yield: 79.6%. ^1^H NMR (600 MHz, DMSO-d_6_) δ 8.58 (s, 1H, NH-H), 8.01 (s, 1H, NH-H), 7.68 (d, J = 12.0Hz, 1H, CH-H), 7.40–7.28 (m, 6H, Ar-H), 7.05 (d, J = 12.0Hz, 1H, Ar-H), 6.57 (s, 1H, Ar-H), 5.65 (s, 2H, CH_2_-H), 1.64 (s, 6H, 2CH_3_-H). ^13^C NMR (150 MHz, DMSO-d_6_) δ 154.3, 154.1, 140.3, 135.6, 133.3, 133.3, 131.8, 130.7, 130.4, 128.7, 123.0, 122.3, 119.8, 112.6, 87.7, 53.2, 50.6, 40.5, and 28.8.

***1-(2-(1-(3-bromobenzyl)-1H-1,2,3-triazol-4-yl)propan-2-yl)-3-(4-bromophenyl)urea (3j)***. White solid. LC-MS(ESI): m/z 492.00 (M + H)^+^. Yield: 72.8%. ^1^H NMR (600 MHz, DMSO-d_6_) δ 8.58 (s, 1H, NH-H), 8.07 (s, 1H, NH-H), 7.54–7.52 (m, 2H, CH-H, Ar-H), 7.46–7.43 (m, 2H, Ar-H), 7.36–7.34 (m, 2H, Ar-H), 7.31–7.28 (m, 2H, Ar-H), 6.57 (s, 1H, Ar-H), 5.58 (s, 2H, CH_2_-H), 1.63 (s, 6H, 2CH_3_-H). ^13^C NMR (150 MHz, DMSO-d_6_) δ 154.3, 154.2, 140.3, 139.3, 132.0, 131.7, 131.4, 131.4, 131.1, 127.4, 122.3, 122.1, 120.7, 119.8, 112.6, 52.2, 50.6, and 28.8.

### 5.4 IDO1 enzymatic inhibition assay

To assess the inhibitory effect of the designed compounds against IDO1, Hela cells were seeded at 50,000–60,000 cells per well in 100 μL of complete Dulbecco’s modified eagle medium growth medium in a 96-well plate and incubated for 12–18 h. On the second day, diluted inhibitor at a final concentration of 100 ng/mL human IFN-γ was added (100 μL per well) and incubated at 37°C with 5% CO_2_ for 18 h. On the third day, 140 μL of medium was transferred into a new 96-well plate, and the protein was precipitated with 10 μL of 6.1 N trichloroacetic acid (CCl_3_COOH) at 50°C for 30 min. The plate was then centrifuged at 2,500 rpm for 10 min, and 100 μL of supernatant was transferred to another 96-well plate and mixed with 100 μL of 2% (w/v) p-dimethylaminobenzaldehyde in CH_3_COOH. The plate was incubated at 25°C for 10 min, and the yellow color derived from kynurenine was measured at 480 nm using a microplate reader (PE, United States). Inhibition curves with IC_50_ values were generated using Prism 6.0.

### 5.5 Molecular docking

Docking experiments were conducted using Schrödinger (Schrödinger LLC 2015; United States). The 4PK5 complex system with Amg-1 as the co-crystal ligand was chosen for the study. The protein was processed and optimized using the Protein Preparation Wizard, which involved hydrogenation of the protein, assignment of bond orders, creation of zero-order bonds with metals, creation of disulfide bonds, removal of water molecules larger than 5 Å from het groups, structural optimization, and energy minimization. The ligands were constructed using ChemDraw and optimized using the LigPrep module under the condition of pH 7.0 ± 2.0 for protonation and generation of stereoisomers. Molecular lattices centered on Amg-1 were generated, and metal coordination constraints centered on heme iron were applied. Docking was performed using SP precision.

## Data Availability

The original contributions presented in the study are included in the article/Supplementary Materials, further inquiries can be directed to the corresponding author.
